# Pathological Neural Attractor Dynamics in Slowly Growing Gliomas Supports an Optimal Time Frame for White Matter Plasticity

**DOI:** 10.1371/journal.pone.0069798

**Published:** 2013-07-26

**Authors:** Krisztina Szalisznyo, David N. Silverstein, Hugues Duffau, Anja Smits

**Affiliations:** 1 Department of Neuroscience, Neurology, Uppsala University, Uppsala, Sweden; 2 PDC Center for High Performance Computing and Department of Computational Biology, KTH Royal Institute of Technology, Stockholm, Sweden; 3 Stockholm Brain Institute, Karolinska Institute, Stockholm, Sweden; 4 Department of Neurosurgery, Gui de Chauliac Hospital, CHU Montpellier, Montpellier University Medical Center, Montpellier, France; 5 Department of Biophysics, Wigner Research Centre for Physics, Hungarian Academy of Sciences, Budapest, Hungary; McGill University, Canada

## Abstract

Neurological function in patients with slowly growing brain tumors can be preserved even after extensive tumor resection. However, the global process of cortical reshaping and cerebral redistribution cannot be understood without taking into account the white matter tracts. The aim of this study was to predict the functional consequences of tumor-induced white matter damage by computer simulation. A computational model was proposed, incorporating two cortical patches and the white matter connections of the uncinate fasciculus. Tumor-induced structural changes were modeled such that different aspects of the connectivity were altered, mimicking the biological heterogeneity of gliomas. The network performance was quantified by comparing memory pattern recall and the plastic compensatory capacity of the network was analyzed. The model predicts an optimal level of synaptic conductance boost that compensates for tumor-induced connectivity loss. Tumor density appears to change the optimal plasticity regime, but tumor size does not. Compensatory conductance values that are too high lead to performance loss in the network and eventually to epileptic activity. Tumors of different configurations show differences in memory recall performance with slightly lower plasticity values for dense tumors compared to more diffuse tumors. Simulation results also suggest an optimal noise level that is capable of increasing the recall performance in tumor-induced white matter damage. In conclusion, the model presented here is able to capture the influence of different tumor-related parameters on memory pattern recall decline and provides a new way to study the functional consequences of white matter invasion by slowly growing brain tumors.

## Introduction

Gliomas are primary brain tumors derived from glial cells and consist of high-grade and low-grade gliomas [1]. The majority of gliomas arise from anterior subcortical brain structures, with predominance for the frontal and temporal lobes [2]. Low-grade gliomas (LGG) in adults have malignancy grade II according to the WHO classification and are characterized by extensive invasion but only a low proliferation rate [1], [3]. In spite of their “benign” behavior at onset, LGG are precancerous tumors with a potential for transformation into high-grade gliomas.

Seizures are the most frequent presenting symptom in patients with LGG [4]. Focal neurological signs and major cognitive deficits are uncommon for LGG at onset but occur in high-grade gliomas, which typically cause focal brain damage and compression of normal brain tissue by rapid and destructive growth. In addition, anxiety, depression, and fatigue are commonly seen in patients with brain tumors [5]. Mood changes in brain tumor patients are related to tumor location [6]. Additional factors that contribute to changes in cognitive and emotional function in patients with LGG are seizures, antiepileptic drugs, surgery, radiotherapy, chemotherapy and psychological reactions to the disease [7], [8].

Sequential MRI studies of LGG have shown that the growth of the diameter of the bulky tumor mass before malignant transformation is apparently linear over time [9]. In parallel with a continuous expansion over time, LGG progressively migrate along the white matter pathways. It is generally difficult to define the degree of tumor invasion into the surrounding brain tissue, since the mobile tumor cells that spread into the adjacent brain tissue cannot be detected by current MRI techniques [10]. A modeling study predicted that many newly produced tumor cells migrate into the surrounding parenchyma but their density does not reach the minimal threshold required to appear on MRI [11]. Indeed, because of anisotropic migration along white fibers, LGG frequently exhibit a complex configuration with a roughly ellipsoid shape, mimicking the subcortical pathways [3]. The invasion rate is estimated to be about five times higher in the white matter than in the grey matter [12].

The invaded brain tissue is often regarded as a homogeneous and passive region modified by the tumor in a unidirectional manner [13]. However, reciprocal processes and multiple sets of active and passive mechanisms are present in the invaded tissue [14]. Brain plasticity most commonly refers to changes in the strength of synaptic connections, leading to functional or morphological reorganization [14], [15]. Due to brain plasticity, even a large tumor mass may not cause neurological symptoms. Consequently, patients with LGG can undergo massive cerebral resections and it is well known that neurological recovery in these patients is significantly better than after acute brain injuries [16], [17]. Thus, functional recovery is directly influenced by the kinetics of the lesion, and slow versus acute lesions involve very different patterns of reorganization. The neural counterpart of this disparity is still poorly understood. Postlesional recovery in acute lesions has been shown to involve mainly ipsilesional structures adjacent to the injury, while slowly growing lesions utilize both adjacent and distant areas in the ipsi- and contralesional hemisphere [16].

Apart from the more frequently studied grey matter plasticity, subcortical connectivity and organization of white matter tracts are crucial for reorganization. However, the capacity to build new structural connectivity leading to functional recovery has not been demonstrated yet in humans [18]. The possible mechanisms through which white matter reorganization occurs include unmasking of perilesional latent networks, recruitment of accessory pathways, introduction of additional relays within the circuit, involvement of parallel long-distance association pathways [18]. Subcortical pathways seem to have a crucial role in shaping cortical reorganization following perturbations of normal function [19]. For example, following surgery in language circuits, plastic reorganization is possible only if the subcortical connectivity is intact [20]. In general, white matter plasticity is considerably more limited than grey matter plasticity.

Fiber tracts around the insula are susceptible to glioma invasion and the uncinate fasciculus (UF) is a common location for limbic glioma invasion [3]. The UF hooks around the limen insulae to link the temporal pole (TP) and the lateral orbitofrontal cortex (OF). In this study, we present a computational model where we used the anatomical characteristics of the UF to study the functional consequences of tumor invasion in white matter tracts. Tumor-induced structural changes were modeled by altering the white matter connections of this bundle, mimicking the biological heterogeneity of LGG. The network performance was quantified by comparing memory pattern recall and the plastic compensatory capacity of the network was analyzed. We show that the model is able to predict the functional consequences of white matter damage by slowly growing tumors.

## Materials and Methods

### The uncinate fasciculus (UF)

The UF is the most rostral temporal lobe fiber bundle. It interconnects the inferior frontal and the anterior temporal lobes and provides an afferent sensory route for prefrontal cognitive functions [21]. The human UF is bidirectional with an average length of 45 mm (range 40–49 mm) and is asymmetrical, being larger and containing more fibers in the right than in the left hemisphere [21]. The UF is thought critical for processing novel information, understanding emotional aspects of the nature of sounds and for the regulation of emotional responses to auditory stimuli. UF connections contribute to the interaction between emotion and cognition. The right and left UF are amongst the few resectable white matter pathways, according to the intraoperative electrical stimulation map [22].

### Neural attractor model

**Figure 1:**
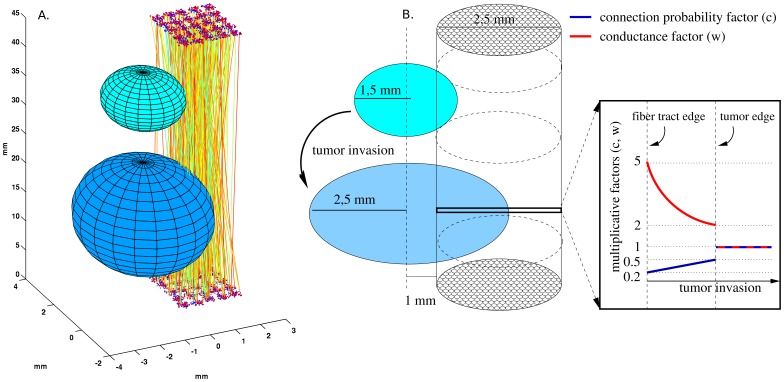
Schematic representation of tumor growth and the two neocortical patches with interconnecting fibers. A) The patches contain 5×5 hypercolumns and are interconnected by bidirectional fibers, which are invaded by a tumor. The tumor growth is represented by two different sizes. B) The center of the tumor was assumed to be outside of the fasciculus. The tumor invasion expands into the fiber, such that the center of the tumor remains fixed. Due to the invasion, the multiplicative connection probability factor (blue line) changes, such that the connection probability decreases closer to the tumor center and increases towards the tumor edge. With compensatory plasticity, the multiplicative conductance factor (red line) changes as well, such that closer to the tumor center, the conductance increases, and towards the tumor edge it decreases. In this example, the multiplicative value of the conductance and connection probability was conserved with a plasticity factor of 1. Outside the tumor, both the connection probability and the conductance were unaffected.

**Figure 2:**
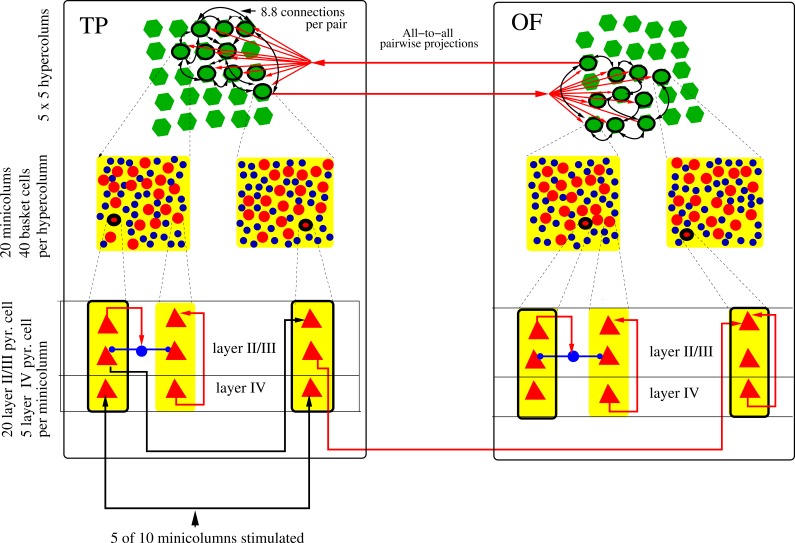
Schematic drawing of the neocortical minicolumns and hypercolumns used in the simulations, at three levels of abstraction. Both the temporal pole (TP) patch and the lateral orbitofrontal (OF) patch contain 5×5 hypercolumns (green). Each minicolumn contains 20 layer II/III pyramidal cells and 5 layer IV pyramidal cells (not all represented in the figure). Layer IV pyramidal cells have feed-forward excitatory projections to layer II/III pyramidal cells and layer II/III pyramidals have recurrent connections. Each hypercolumn contains 40 basket cells (blue), which provide lateral inhibition between minicolumns (blue arrows). Out of 25 hypercolumns, 10 are randomly selected, but spatially clustered for each memory pattern (black ring), and from each selected hypercolumn, a single minicolumn is randomly chosen. These 10 minicolumns have all-to-all excitatory synaptic connectivity with an expectation of 8.8 connections between any pair of minicolumns. In this figure, only some of the 90 connection pairs are shown. While synaptic connections are unidirectional, they occur in both directions for each minicolumn pair, so they are abstractly displayed as bidirectional. Each memory pattern local to one patch is connected to a corresponding one in the other patch with excitatory projections (red arrows). The pattern projections are all-to-all between the patches (in both directions) and have 1 fiber between any pair of minicolumns. For external stimulation, layer IV pyramidal cells in 5 random minicolumns out of the 10 in a selected memory pattern are stimulated in the TP patch (black arrow). This can activate a local attractor, which then propagates to the OF.

We computationally modeled a bidirectional fiber tract between two neocortical patches, together with an invading tumor (Figure 1). A stimulated source patch represents the temporal pole (TP) while the destination patch represents the lateral orbitofrontal cortex (OF). The length of the fiber tract was set to 45 mm. The neocortical patch model is similar to as described in [23], including the neural model descriptions with equations, but with some modifications, as described in the supporting information (Text S1). Each cortical patch was modeled as a 5×5 square of hypercolumns (Figure 2), containing a total area of 2.5×2.5 mm, with 13,500 neurons. Each hypercolumn contained 20 minicolumns, each with 20 layer II/III pyramidal cells and 5 layer IV pyramidal cells. Each hypercolumn also included 40 basket cells, which provided lateral inhibition in layer II/III (Figure 2). Both the number of minicolumns per hypercolumn and the number of neurons in layer II/III of a minicolumn were subsampled by a factor of around 5. In an *x-y* plane, with each coordinate having a range of 0 to 2.5 mm, hypercolumns were separated by 500 µm and minicolumns and basket cells were randomly positioned within each of them. The minimum distance between pairs of minicolumn centers was 30 µm. Between pairs of basket cells, or between basket cells and any minicolumn centers, the minimum distance was 15 µm. For each minicolumn, pyramidal cells in layer II/III and layer IV were placed along a *z* axis. The pyramidal cells had compartments for the soma, initial segment, a basal dendrite and apical dendrite, while the basket cells had compartments for the soma, initial segment and basal dendrite.

**Figure 3:**
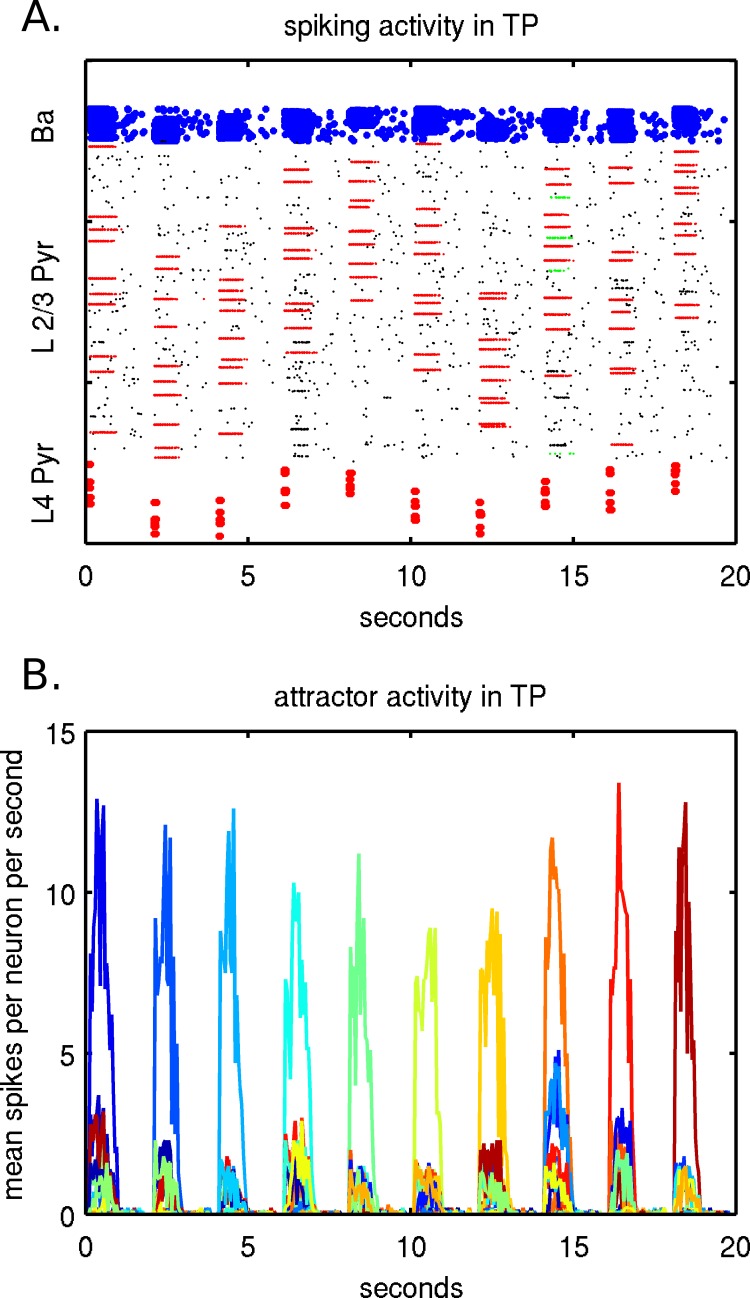
Example of neural activity in TP during recall of 10 memories, in 2 different representations (A,B) of the same simulation. A memory recall is performed every 2 seconds when layer IV pyramidal cells in 5 minicolumns are stimulated. A) Shows spiking activity of individual neurons in TP with stimulated layer IV pyramidal cells on the bottom, activated minicolumns in layer II/III in the middle and basket cells on the top. Five corresponding minicolumns in layer II/III initially activate and after the pattern completes, 10 minicolumns are activated, which can be seen individually as lines. B) Shows attractor activity of layer II/III pyramidal cells fromactivated memory patterns. The spiking activity within each memory pattern is averaged over a sliding 50 ms window . The 50 stored memory patterns are represented by different colors along a color spectrum. Memory patterns are tested and recalled sequentially and the corresponding attractor memory dominates, with weaker activity present due to memory pattern overlaps.

The Hodgkin-Huxley formalism was used for all neurons and included calcium dynamics (see supporting information Text S1). Synaptic channels were modeled for AMPA/Kainate, NMDA and GABA_A_, and included synaptic depression. Synaptic noise was simulated with 300 Hz Poisson processes and applied spiking events to excitatory, AMPA like synapses on dendrites of the layer II/III pyramidal and basket cells (see supporting information Text S1). The long-range connectivity between hypercolumns within each cortical patch was generated separately with a different random seed and stored 50 static, randomly selected, sparse and overlapping memory patterns. Each stored memory pattern was a neural assembly formed with long-range synapses on layer II/III pyramidal cells, between randomly selected minicolumns in 10 of 25 different hypercolumns, with one minicolunm chosen per hypercolumn (Figure 2). The memory patterns were spatially localized within the patches by randomly choosing hypercolumns, such that the distance from the sample centroid was chosen from a Gaussian distribution with σ = 707 µm. Of the minicolumns selected for each memory pattern, synapses were created within the patch based on an expectation of 8.8 connections between each pair of minicolumns. Each connection was a terminal cluster having one sending pyramidal cell from the sending minicolumn and having an expectation of 10 synapses total on pyramidal cells of the receiving minicolumn. The model was tuned for pattern completion, such that when 5 of 10 minicolumns of a memory pattern were stimulated, the full pattern becomes active. The 8.8 expectation takes into consideration the long-range pyramidal-pyramidal synaptic conductance values. If the expectation is much lower, the stored memory pattern will not become an attractor when stimulated. If the expectation is higher, when the attractor activates, it will sustain longer, and at high enough values, would not be physiologically realistic. A high expectation may also cause spontaneous attractor activation. We define an attractor as when a memory pattern becomes active and is sustained with recurrent activity. An attractor can become locally active within a patch and globally active across both patches. An attractor dwell time is typically 700–1000 ms. Figure 3 A&B shows an example of spiking and attractor activity in TP during memory recall under normal conditions, without the presence of a tumor. In order to test memory recall, a memory pattern must be partially stimulated, since at baseline noise levels without stimulation, an attractor will not become active. However, with sub-threshold stimulation and additional noise, attractors can activate. Spontaneous activity is also possible without stimulation if the noise level is high enough. Spurious attractors do not occur under normal circumstances, because the total of 50 memory patterns is below the storage capacity of the network and memory pattern overlaps are generally not high enough. This changes however, when the tumor damages projections, which can cause spurious activity. Pattern completion within a patch can take around 50 ms, due to propagation speeds within the patch (0.5 m/s) and microcircuit dynamics. For instance, pyramidal cells must receive multiple spikes before they will fire. With a fiber tract conduction velocity of 2 m/s and a fiber tract length of 45 mm, it takes a minimum of about 50 ms round-trip time for neural activity to propagate, which is on a similar time-scale as pattern completion within a patch.

Bidirectional fiber tract projections with symmetrical conductances between the neocortical patches connected individual memory patterns (sets of minicolumns) in the source patch (TP) with corresponding individual memory patterns in the destination patch (OF). This way, a memory pattern is both locally represented within the patches and globally represented across the patches with the memory pattern projections (Figure 2). Before perturbations from the tumor, the projections between patches in each direction are all-to-all between minicolumns of the corresponding memory patterns, with an expected value of 1 fiber between each pair of minicolumns. Thus, in a patch, one minicolumn in each memory pattern projects to an average of 10 minicolumns in the other patch. The fiber tract was modeled as synaptic connections with delays and varying conductance values, but not as axonal compartments. The fiber tract connecting the patches had about 10,000 fibers for both directions, with a density of about 1600 fibers per mm^2^, subsampled by a factor of around 70. The projection subsampling was a reflection of the subsampling of the cortical patches. According to previous studies, there are around 110,000 axons per mm^2^ in the UF [22]. Each fiber targeted layer II/III pyramidal cells in a single minicolumn of the receiving patch, with a terminal cluster of 10 glutaminergic synapses on average. Fibers in the tract connecting the two cortical patches had a transmission speed of 2 m/s.

### Quantifying memory recall

Stored memory pattern completion was examined as a function of varying fiber tract connectivity damage between the two patches. The effect of connection probability loss was analyzed, while varying the compensatory synaptic conductance when different tumor densities caused totally diffuse or spatially localized damage. The effect of increasing the synaptic noise level was also studied. The correct pattern was considered completed when the cumulative activity of all the neurons in the stimulated pattern was above threshold in both patches. The total spiking activity was measured in layer II/III pyramidal cells in minicolumns of individual patterns. For the activity to be above threshold in the stimulated pattern during a memory recall interval, the total number of spikes should be above a certain value. This threshold was calibrated to distinguish between the spiking activity within the 5 stimulated minicolumns and the spiking activity, which occurs when the pattern completes and the additional 5 minicolumns become active as well. Three different error types were detected: 1) no pattern completion; 2) completion with spurious activity (correctly activated attractor shifts to a different pattern in either patch); 3) activation of incorrect pattern. Calcium-dependent dynamics and synaptic depression of a correctly activated memory pattern causes adaptation and termination of attractor activity, but if there is overlapping connectivity and activity or noise, another attractor can become active. If an incorrect memory pattern is activated in conjunction with the correct one, the recall is considered spurious. A memory recall error is defined to occur when the stimulated memory pattern does not become active, but others do. To activate an attractor memory, 5 of 10 minicolumns were randomly selected and the layer IV pyramidal cells in these minicolumns were stimulated for 60 ms, which consisted of pulses at 14 ms intervals on average. From this, each stimulated layer IV pyramidal cell received a maximum of 4 input pulses. On stimulated minicolumns, all 5 layer IV pyramidal cells received input pulses. If the activation spreads to all 10 minicolumns in a memory pattern, the memory completes and the attractor memory is activated. In this way, activation of an attractor memory in the source patch can propagate to the destination patch and vice versa. It is also possible that the wrong memory pattern can activate in the destination patch, which could then recurrently activate the wrong memory pattern in the source patch as well.

### Modeling compensatory plasticity

Several computational experiments were performed to explore the impact of connection loss in the UF and compensatory mechanisms. Prior to simulating tumor effects, a sensitivity analysis was performed on connection probability and synaptic conductance, using multiplicative factors. In the simulated lesion experiments, a plasticity factor was introduced (see supporting information Text S1), based on a compensatory increase in conductance for connection loss; a plasticity factor of 0 means no compensatory increases in conductance, while a plasticity factor of 1 means total compensation, such that the multiple of the connection probability and conductance factor was conserved. For the sensitivity analysis, memory patterns were randomly generated without spatial localization.

### Modeling tumor invasion

When simulating tumor effects, the center of the tumor was always outside the white matter fasciculus (Figure 1A and Figure 1B), with a minimum distance of 1 mm, so no tumor effects were applied outside the fiber tract. The tumor invasion was modeled such that the connection probability dropped linearly from 0 at the tumor center to 50% (less dense) or 75% (more dense) loss of the original connection probability at the tumor edge (Figure 1B). With compensatory plasticity, synaptic conductance was increased with connection loss, increasing within the fiber tract from the tumor edge towards the tumor center. Two different simulation sets were performed in experiments with additional noise levels, one with and one without plasticity. In the simulations without plasticity, we incrementally increased the noise level in any projections intersecting the tumor in both directions (from TP to OF and from OF to TP). At the center of the tumor outside the fiber tract, the noise level would have increased by 0–150%, which linearly decreased to 0% at the tumor edge, inside the fiber tract. Thus, the closer the fiber tract was to the tumor center, the higher the increased noise. In the simulation sets with plasticity and variable noise, the noise levels were increased by 25% or 50% at the center of the tumor (but outside the fiber) and linearly decreased to 0% (baseline) at the tumor edge. This was intended to represent additional synaptic noise induced from tumor pressure on the fiber tract. In the sensitivity analysis, 10 of 50 stored memory patterns were tested for memory pattern recall, while in all other experiments, 30 of 50 stored memory patterns were tested.

### Simulation methods

Simulations were performed using the Parallel NEURON simulator [24] version 7.2, running on a cluster of AMD/Opteron-based Supermicro motherboards and connected with an InfiniBand interconnect. Model geometry and synaptic connectivity files were constructed with Matlab, and then imported into the NEURON simulation code, which was written with the Hoc interpreter and NMODL. Separate simulations were performed for individual data points in each of the plots. For the sensitivity analysis, parameters were varied for the connection probability factor and conductance factor. In other simulations, parameters were varied for the tumor radius, tumor density, plasticity factor, noise level and conduction speed. Individual simulations utilized 168 MPI processes running on 168 cores across 7 compute nodes. Each simulation had 10 or 30 memory pattern recall tests within it, and each memory pattern recall test was for 1.8 seconds of cortical activity, followed by a 200 ms barrier. During this barrier time, synaptic depression variables were reset. The simulation time-step was 50 µs.

### Ethics Statement

Written informed consent as outlined in the PLOS consent form was obtained from the patient presented as a clinical case report to publication of his case details.

## Results

### A sensitivity analysis of altered connection probability and synaptic conductance

**Figure 4:**
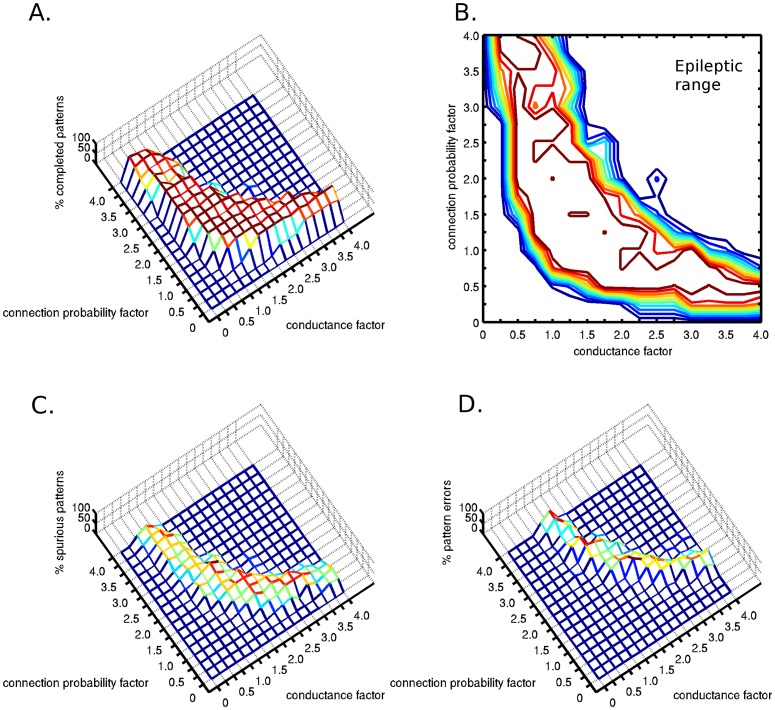
Two-dimensional sensitivity analysis of the attractor dynamics with respect to pattern completion. The connection probability and the synaptic conductance parameter space were examined assuming complete compensatory plasticity (plasticity factor of 1). Diffuse tumor invasion was represented by the connection probability factor <1. A) The percentage of completed patterns were plotted in two dimensional space. B) Contour plot of the completed patterns. C) Percentage of activated spurious patterns. D) Percentage of false patterns, without the activation of the stimulated correct pattern. At even higher connection probability and conductance values, epileptic neural activity was observed.

We investigated the consequences of decreased connection probability in parallel with increased synaptic conductance, while maintaining constant postsynaptic depolarization within activated memory patterns. The assumption behind these simulations was that the decreased connection probability is compensated by increased synaptic conductance through plastic modifications. This allows the neurons to adapt to specific average input levels, and promote network stability. We found that at low connection probabilities, low synaptic conductance values are not sufficient for pattern completion and higher synaptic conductances were needed to complete the stimulated patterns (Figure 4A and Figure 4B). On the other hand, low synaptic conductance values required higher connection probabilities for pattern completion (Figure 4A and Figure 4B). The probability of a completed attractor memory has a plateau regime in a 2-D parameter space in the shape of a rectangular hyperbola. It is important to note that in the regime with lower connection probabilities, the higher conductance values were able to compensate for the function only in a narrower parameter regime, thus the contour-plot figure is not symmetric (Figure 4B). This is likely because lower connection probabilities eventually disallow complete pattern representations whereas lower synaptic conductances do not. At high parameter values, the correctly completed pattern probability drops due to the increased excitability of the network, which results in false, unstimulated patterns becoming active. The probability of spurious activity increases with increased connection probabilities and with increased synaptic conductance values (Figure 4C). As the connection probability and conductance values increase, spurious activity appears until finally errors occur (Figure 4D), while the stimulated memory pattern does not complete (Type 3 error). At even higher connection probability and synaptic conductance regimes, epileptic neural activity was observed, with persistent high frequency firing. This set of simulations captured the case of diffuse white matter damage, which occurs in diffusely growing tumors.

### Altering tumor size and tumor configuration

**Figure 5:**
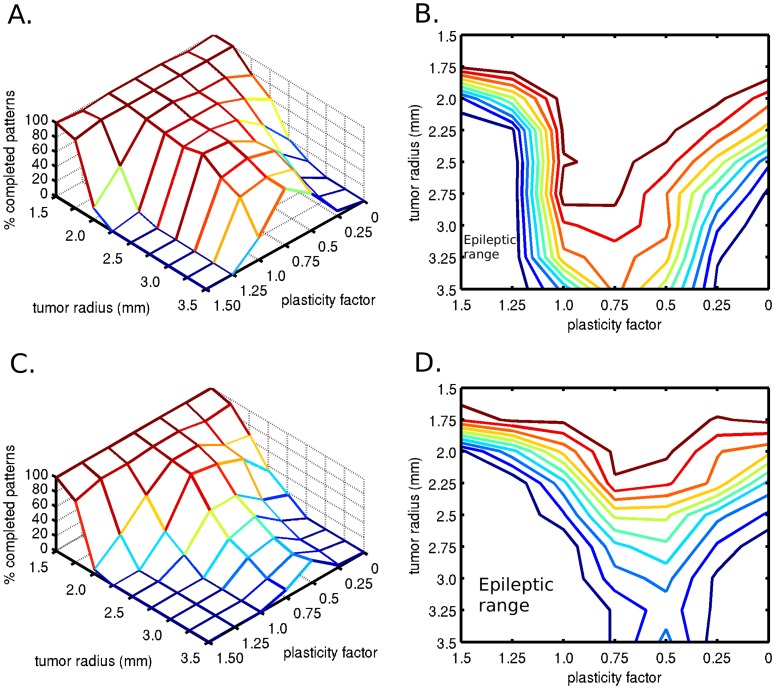
Effect of tumor size on attractor dynamics. A) Influence of tumor radius on the percentage of completed patterns, when connection probability loss at the tumor edge is 50%. In the presence of optimal compensatory synaptic plasticity, the memory pattern completion is better preserved than without plasticity. B) With the same connection probability loss as figure 4A, contour plot showing an optimal compensatory synaptic plasticity, which is capable of preserving the pattern completion function even with larger tumor size. C) Effect of tumor size on attractor dynamics in case of a denser tumor, when the connection probability loss at the edge of the tumor was 75%. Again, there exists an optimal compensatory synaptic plasticity, which is capable of preserving partial memory recall even with larger tumor size, but with a sharper functional decline. D) With the same connection probability loss as in Figure 4C, the contour plot demonstrates the optimal plasticity factor.

In this set of simulations, a topographic representation of tumor invasion was assumed. We found a sharp decrease in the ability of pattern completion with tumor growth (Figure 5A). Plastic conductance increase was able to compensate for drop in pattern completion ability (Figure 5A). The model predicts an optimal conductance regime, suitable to compensate for the tumor-induced performance loss (Figure 5B). This regime is independent of tumor size. Next, with the same simulation protocol, a denser tumor was examined (Figure 5C and Figure 5D). Simulation results suggest a steeper performance loss than in case of a less dense tumor. Also, the performance of the network degraded faster as the tumor size increased, compared to a less dense tumor (Figure 5C versus Figure 5A). An optimal compensatory conductance regime was found in both tumor densities, which was independent of the tumor size. It is interesting to note that the simulation results suggest different optimal plasticity regimes with the different tumor densities. Thus, tumor density appears to change the optimal plasticity regime, but tumor size does not.

### Altering noise level

**Figure 6:**
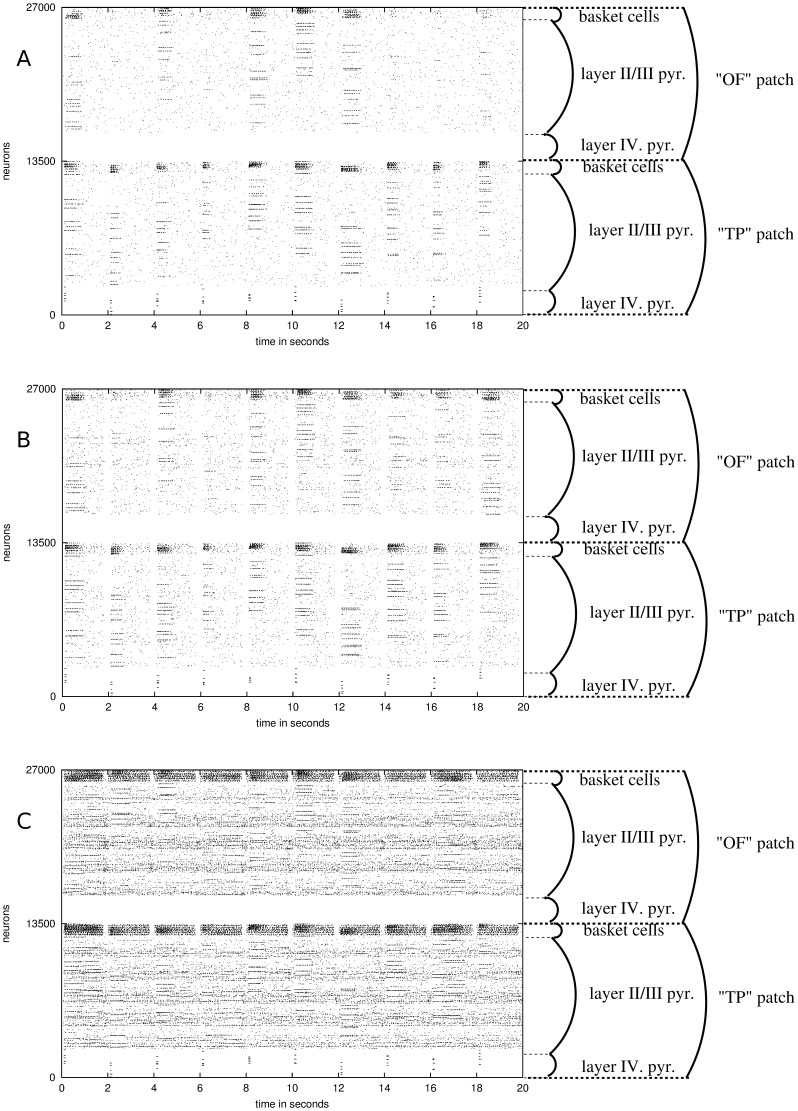
Effect of increased synaptic conductance noise on memory pattern transmission and recall across the UF. The synaptic conductance noise was increased from A to C with the presence of a tumor of radius 2.5 mm. This figure was based on the same data presented in Figure 7A and 7B. The noise was increased based on the fiber proximity to the tumor. A stimulated source patch represents the temporal pole (TP) while the destination patch represents the lateral orbitofrontal (OF) cortex. On each plot, each column occurring every 2 seconds represents a memory pattern recall test, with activated (from bottom) layer 4 pyramidals, layer II/III pyramidals and layer II/III basket cells. Each line within the layer II/III pyramidals shows an active minicolumn within a stored memory pattern. A) Noise at baseline. B) Noise increased up to 15% based on proximity to tumor (would be 25% at tumor center). C) Noise increased up to 45% based on proximity to tumor (would be 75% at tumor center).

**Figure 7:**
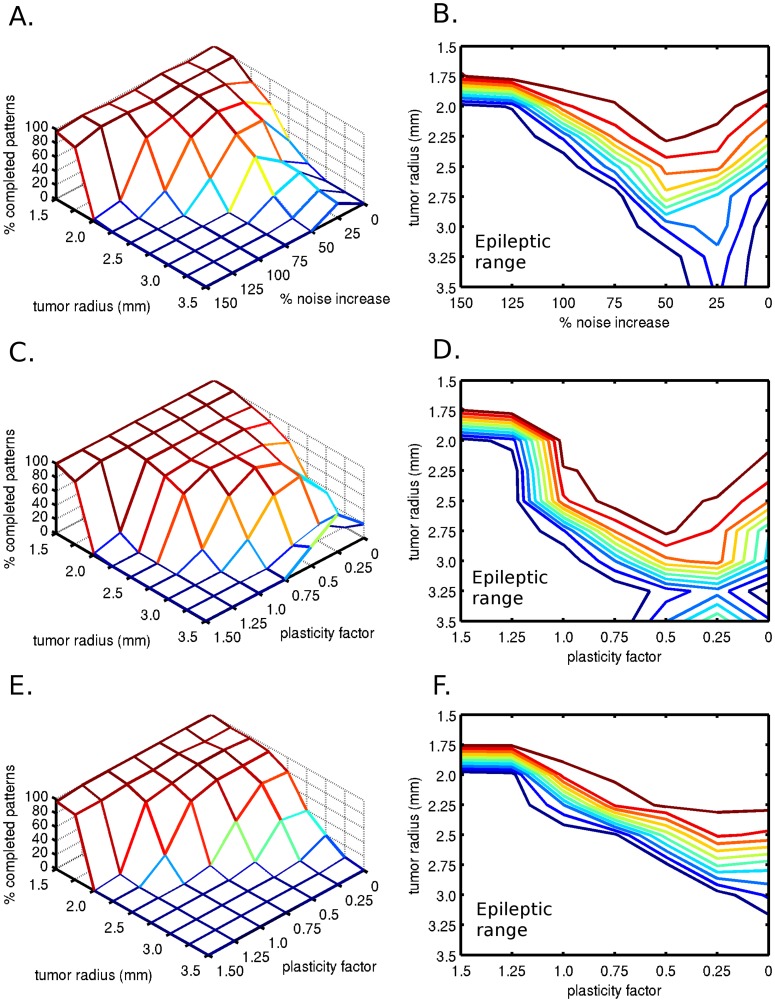
Effect of additional synaptic conductance noise on memory pattern completion. A) Effect of increasing noise levels and tumor growth on the percentage of completed patterns, with a connection probability loss of 50% at the tumor edge. B) Contour plot of A, showing an optimal noise level for pattern completion. C) Effects of plasticity and tumor size with up to 25% additional noise on memory pattern completion. The additional noise was linearly increased based on proximity from the tumor center, where it would be 25% higher than baseline, with a connection probability loss at the tumor edge of 50%. D) Contour plot of C, showing the percentage of completed patterns. E) Effects of plasticity and tumor size with up to 50% additional noise on memory pattern completion. The additional noise was linearly increased based on proximity from the tumor center, where it would be 50% higher than baseline, with a connection probability loss at the tumor edge of 50%. F) Contour plot of E, showing the percentage of correctly completed patterns.

To simulate the effects of increased noise due to pressure and micro-environmental changes by tumor invasion, we decreased the conductance of projections in both directions (from TP to OF, and from OF to TP) and increased the synaptic noise levels on layer II/III pyramidals on both the TP and OF patches, based on distance from the tumor. An example of this can be seen in Figure 6, which shows three different noise levels with a 2.5 mm radius tumor, starting with baseline (Figure 6A). The noise was increased 15% and 45% where the fiber was closest to the tumor center, and decreased to 0% (baseline) at the tumor edge. See supporting information (Text S1) for details. This example demonstrates that with increased noise (Figure 6B), subthreshold activity can become active and cause memory pattern completion. With an increased noise level, the attractor dwell time also increased, both at the source as well as at the destination patch. With an even higher increased noise level, memory pattern recall degrades (Figure 6C). Some stimulated memory patterns complete in both patches while others do not. When memory patterns and corresponding fibers are clustered more closely to the tumor, due to spatial locality of the memory patterns, the memory recall test is more likely to fail. We also discovered that an optimal noise level exists that improves the stored memory recall performance (Figure 7A and Figure 7B).

### Altering noise level with plasticity

In the final set of simulations with both noise and plasticity, with an increased noise level of 25% (Figure 7C and Figure 7D), the optimal plasticity factor moved towards lower plasticity values (comparing Figure 5A, Figure 5B with Figure 7C, Figure 7D). The increased noise level of 50% at a 0 plasticity factor improved the network performance (comparing Figure 5A, Figure 5B with Figure 7E, Figure 7F), but at higher noise factors the optimal plasticity regime disappeared (Figure 7E and Figure 7F).

### Clinical case report

**Figure 8:**
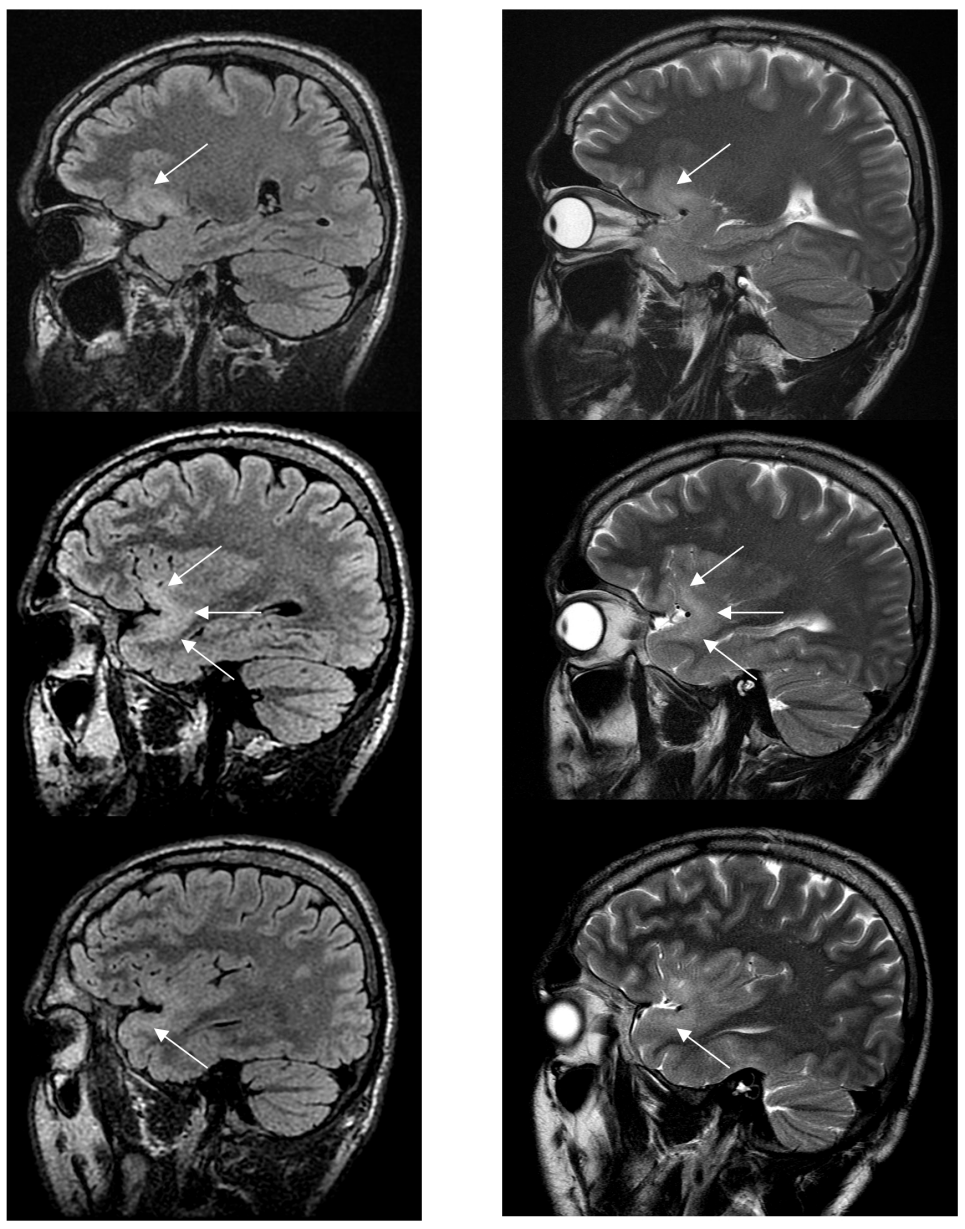
Sagittal FLAIR-weighted MRI (left) and sagittal T2-weighted MRI (right) performed in a 30-year-old right-handed asymptomatic patient who participated in a research protocol. This MRI shows a left WHO grade II glioma involving the uncinate fascicle (white arrows), from the orbitofrontal part (upper) to the temporopolar part (lower) through the temporal stem (middle).

We present a 30-year-old right-handed asymptomatic patient including normal cognitive function with an incidental LGG who participated in a research protocol (Figure 8). The sagittal FLAIR-weighted MRI (left) and sagittal T2-weighted MRI (right) of this patient showed a left LGG involving the UF (white arrows), from the orbitofrontal part (upper) to the temporopolar part (lower) through the temporal stem (middle), at the time point of radiological diagnosis. During the next 10 months, this patient did not receive treatment and the tumor volume increased from 4.5 cc to 8 cc, i.e. with a median velocity of diametric expansion of around 4 mm/year, in agreement with the natural history of LGG [3]. His cognitive assessment was normal but he experienced an inaugural seizure (partial uncinate epilepsy) at 11 months after radiological diagnosis. He benefited from awake surgery at this time point and showed no functional loss after complete tumor resection.

## Discussion

Computational models contain substantial simplifications of biological reality, and predicting consequences of brain tumors by mathematical models may seem daunting. However, the power and possibility of modeling lies in teasing apart the different components and to capture key pathophysiological properties of complex diseases. *In silico* research therefore represents a novel approach to study the effects of tumor growth that complements traditional methods. Most modeling efforts have targeted brain tumor profiles at the macroscopic level, based on imaging results [25]. To our knowledge, only one study has analyzed the functional consequences of tumor-induced damage, which was the goal of the present work [17].

We found evidence in our model for an optimal plasticity regime for synaptic conductance that allows compensation of the connection probability loss. By further increasing the compensatory synaptic conductance, spurious patterns appear, in parallel with the stimulated ones. Finally, false patterns are activated without the stimulated ones and epileptic activity emerges. From a clinical point of view, these experimental findings suggest that once seizures occur in LGG, the final stage for compensatory reorganization has been reached and plasticity is already at the overcompensated stage [26]. The model predicts a decline in memory recall before the epileptic regime is reached. The common clinical experience for patients suffering from LGG is that epileptic seizures are the first symptoms. In many cases patients do not experience cognitive decline or only in a very mild form. Local compensation as studied here takes place before the occurrence of remote recruitments, which are not part of the typical functional network. It is most likely that LGG resections without functional deficits would be impossible with the lack of remote network reorganization [16]. In parallel, we present a case report of a patient with an incidental LGG who experienced an inaugural seizure almost one year after radiological diagnosis. At this time point, his neuropsychological assessment was still within normal limits. He retained normal function after complete tumor resection, suggesting that plasticity mechanisms compensated for the loss of anatomical structures.

Interestingly, the optimal compensatory conductance regime that could compensate for the performance loss was independent of tumor size in our model. Similar predictions were made in case of a denser tumor, but with slightly lower optimal plasticity values compared to more diffuse tumors. These results suggest that it is not only the tumor volume itself, but the configuration of the tumor and the degree of white matter invasion that sets the limits for brain plasticity. In agreement, asymptomatic patients with LGG may have voluminous tumors [27]. Our simulation results suggest that the optimal plasticity factor is dependent on the tumor density, but not on the tumor volume. However, even at an optimal plasticity factor, the network performance is affected by tumor volume.

LGG are diagnosed at various steps along the continuum of their natural course, which is proposed to occur as a three-step process; an initial silent period, followed by a symptomatic period, and a final period of malignant progression [28]. As mentioned, there is a linear expansion of the mean tumor volume diameter during the entire time course prior to malignant progression [9], [29]. Radio- and chemotherapy can induce a temporary slowing down of tumor expansion, which is usually accompanied by improved seizure control [28], [30]. The exact temporal relationship between seizure control and tumor control remains unclear. Recurrent seizures after an initial seizure-free period or late-onset seizures are frequent early signs of tumor progression, but breakthrough seizures may occasionally precede radiological evidence of progressive disease. Our model provides a novel way to study the relationship between neurological function and tumor expansion over time. The clinical application of the model is exemplified by its ability to predict when, during tumor evolution, plasticity can compensate and when epileptic activity can appear in tumors of different sizes and configurations.

Our simulations also predict an optimal noise level that at a certain parameter regime is partially able to restore the weakened white matter connections. In other words, micro-environmental and extra-cellular ion concentration changes around the tumor may increase neuronal excitability. The white matter may have different strategies for retaining normal function during tumor evolution. While increased noise appears beneficial at early stages, lower noise levels are more favorable for plasticity at later stages. A previous computational model demonstrated that high coupling between parallel axons via ephaptic interaction leads to the generation of spurious spikes even in case of myelinated axons [31]. According to a rodent study, the conduction velocity in the thalamocortical white matter in adult mice was estimated to be around 3 m/s [32]. Brain nerve conduction speed measured in human adult visual nerve pathway had a mean velocity value of about 2 m/s [33], which was the conduction velocity chosen in our simulations. In addition, we examined the effects of varied conduction speed. We found that the network performance is partially dependent on the conduction velocity and this dependency is influenced by the tumor size (see supporting information, Figure S1). Further simulations are needed to clarify the specific effects of myelin damage on memory pattern recall in different plasticity regimes and with different tumor configurations.

Future simulations could also dissect the effect of longitudinal tumor growth on epileptogenesis. Earlier studies of posttraumatic brain injury point out the strong association between the spatial pattern of the injured cortex and the propensity for developing posttraumatic seizures [34]. Diffuse head injury was found less prone to develop posttraumatic epilepsy than focal brain trauma. Furthermore, the network that is prone to paroxysmal bursting included a population of cells with a relatively high density of intact neurons [28]. Epileptic activity occurred near the boundary of intact and deafferented areas [34]. Similar findings were observed *in vivo* in experiments with cortical undercut [35]. In our simulations, the compensatory synaptic conductance was applied exclusively in the region affected by the tumor. Further studies will be needed to determine the memory pattern recall of the network with the compensatory synaptic conductance increase outside the damaged region.

Homeostatic plasticity is a synaptic regulation process to counteract changes in activity levels and to maintain overall stability of synaptic strength. Periodic cortical network-wide discharges with bursts of action potentials were observed with a critical threshold for pathological network reorganization, where periods of high activity were disrupted with epochs of relative quiescence in case of severe deafferentation [36]. Although clear critical deafferentation thresholds were not predicted in the current simulations, epileptic activity was observed.

Brain plasticity processes associated with function recovery after focal brain lesion involve not only local areas, but require the reorganization of all brain networks [37]. As mentioned, cognitive impairment in LGG can be traced to a generally mild global, non-focal type of brain dysfunction: memory disturbances, loss of concentration, difficulties with planning and language, and psychomotor slowness [38]. Brain plasticity in slowly growing tumors is paralleled by a marked plastic reorganization of neural networks. Due to reduced global default network mode efficiency, patients with LGG may need to activate multiple areas to perform the required task [39]. Further computational analysis is needed to examine the plastic compensatory potential of more widely distributed neural networks [17]. In this regard, it will be of particular interest to compare brain plasticity induced by tumors with different locations in the brain.

### Study limitations

There are a number of limitations with the present study. First, the exact relationship between the compensatory synaptic conductance in our model and the biological brain plasticity that occurs in LGG is not known. As a second point, the tumor progression time was indirectly incorporated in the model, assuming a decrease in connection probability as tumor evolution occurs. It is important to notice that this parameter cannot be automatically translated into biological time frames. Another limitation of the study is related to the variability in terms of tumor location. We chose to model the UF because of its common site for LGG. The neocortex is divided into functionally specialized areas that are distinguishable based on differences in connectivity and architectural features [40]. However, the intrinsic circuitry and laminar organization are consistent between areas of the adult neocortex, and thus not specific to the UF.

### Conclusions

We present a simplified network model that is able to capture the functional consequences of white matter damage induced by slowly growing tumors and predicts compensatory mechanisms for restoring network function during tumor evolution. Translation of these findings into the clinical situation speaks in favor of early surgery in asymptomatic patients, before the onset of seizures – a time point which, depending on the rate of tumor growth, in essence may already represent the overcompensated stage of brain plasticity.

## Supporting Information

Text S1 Detailed description of the computational model and effects of altered conduction velocity.
(PDF)

Figure S1 The effect of conduction velocity on network performance. Conduction velocity was varied between 0.5 m/s and 8 m/s and the network performance was quantified by the number of completed patterns. Seven different tumor radiuses were examined: 2.5 mm, 2.75 mm, 3 mm, 3.25 mm, 3.5 mm, 3.75 mm, 4.00 mm. With the larger tumor sizes, the network performance increased as the conduction velocity decreased.
(TIF)
