# Supplementary material for: Pathological Neural Attractor Dynamics in Slowly Growing Gliomas Supports an Optimal Time Frame for White Matter Plasticity
Source: PLoS One. 2013 Jul 26;8(7):e69798. doi: 10.1371/journal.pone.0069798 (PMC3724895; doi:10.1371/journal.pone.0069798)
Supplement: Text S1 — Detailed description of the computational model and effects of altered conduction velocity. (PDF) [file pone.0069798.s001.pdf]

SUPPORTING INFORMATION, TEXT S1

Part of the computational model used in this study, including equations and parameters for neurons and synapses, was adapted from Silverstein and Lansner [23].

Effects of altered conduction velocity

With the assumption that the tumor induces conduction velocity change in the white matter, simulations and analyses were performed to examine the effects of the altered conduction velocity. Results were averaged over 4 trials, each having a different set of randomly generated synaptic connection matrices. The conduction velocity of all fibers in the fiber tract was varied between 0.5 m/s and 8 m/s at fixed plasticity factor (1) and with fixed tumor density (0.5) (Figure S1). Different tumor sizes were examined. We found that the network performance appears to be sensitive to conduction velocity in certain cases. With small tumor size (2.5 mm), the network performance appears to be independent of the conduction velocity (Figure S1), likely due to low error rates. With increased tumor size (3.25 mm) and higher error rates, the decreased speed appears to cause a relative increase in network performance, which is likely due to less recurrent error propagation from the degraded fibers.

Plasticity factor and coefficients for connection probability, synaptic conductance

The tumor center was located outside the fiber at a 1 mm distance (Figure 1). The Euclidean distance between each individual fiber from the tumor center was calculated in 2 dimensions (x, y). The 3rd dimension z was assumed to be the same for the tumor center and the mid-point of each individual fiber (located at 22.5 mm on the z axis).

$$d(t,f) = \sqrt{\left(x_t - \frac{x_{TP} - x_{OF}}{2}\right)^2 + \left(y_t - \frac{y_{TP} - y_{OF}}{2}\right)^2}$$

Where the function *d* is the distance between the center of the tumor *t* and a given fiber *f*. Each patch is located within an *x*-*y* plane, with $0 \leq x \leq 2.5$ mm and $0 \leq y \leq 2.5$ mm. (x_{TP}, y_{TP}) represents each fiber *f* minicolumn coordinate in the temporal pole patch, and (x_{OF}, y_{OF}) represents the corresponding fiber minicolumn in the lateral orbitofrontal patch. The coordinates of the tumor center (x_t, y_t) are chosen to be $x_t = 1,\ y_t = -1$. The connection probability coefficient *c* of each fiber from tumor damage was defined according to the following equation and was dependent on the distance *d* of the fiber from the tumor center, the tumor radius *r* and the tumor density *k*.

$$c = \frac{kd}{r}\ where\ d \leq r;\ \ c = 1\ otherwise.$$

The plasticity factor *P* was defined according to how much the conductance *w* is increased to compensate for a drop in connection probability *c* (Figure 1B). In the presence of a tumor:

$$P = cw\ \ where\ c \leq 1\ and\ w \geq 1.$$

Viewed another way, the synaptic conductance coefficient *w* of an individual fiber was dependent on the connection probability coefficient and the plasticity factor.

$$w = \frac{P}{c}$$

Change in noise from the tumor

On apical dendrites of pyramidal cells, a noise synapse receives spikes from a 300 Hz Poisson process. The conductance on the noise synapses can be increased by multiplying it with a noise level coefficient *u*. The noise level coefficient *u* for an individual fiber was dependent on the distance *d* of the fiber from the tumor center, the tumor radius *r* and the noise boost *b* (between 0 and 1.5) according to the following equation:

$u = 1 + \frac{r-d}{r} b \ where \ d < r; \ \ u = 1 \ otherwise.$

Single cell models

The single cell models were described previously in Silverstein and Lansner [23], where the implementation of the Hodgkin Huxley formalism [41] was based on Ekeberg et al. [42]. With the membrane potential V and the Nernst potential E_i for $i \in \{Na, K, Ca, K_{ca}\}$ and given Ohm's law: $I_i = g_i(V - E_i)$ combined with Kirchoff's laws, yields:

$$I_m = C_m \frac{dV}{dt} + g_{NA}(V,t)(V - E_{Na}) + g_K(V,t)(V - E_K) + g_{Ca}(V,t)(V - E_{Ca})$$
$$+ g_{K_{Ca}}(V,t)\left(V - E_{K_{Ca}}\right) + g_L(V - E_L)$$

where g_L is a constant leak conductance. The dynamic conductance $g_i(V,t)$ can be expressed with a gating model for individual ion channels. For modeling the for Na^+ and K^+ ion channel dynamics, Hodgkin and Huxley framework was employed.

$$I_m = C_m \frac{dV}{dt} + \bar{g}_{Na} m^3 h(V - E_{Na}) + \bar{g}_K n^4 (V - E_K) + g_L(V - E_L)$$

where $\bar{g}_i$ with $i \in \{Na, K\}$ is the maximal conductance when a channel is open, and gating variable m is Na^+ channel activation, n is K^+ channel activation h and is Na^+ channel inactivation. The gating variables can be expressed as the following differential equations:

$$\frac{dm}{dt} = \alpha_m (1 - m) - \beta_m m \quad with \quad \alpha_m \frac{A(V - B)}{1 - e^{(B-V)/C}} \quad and \quad \beta_m \frac{A(B - V)}{1 - e^{(V-B)/C}}$$

$$\frac{dh}{dt} = \alpha_h (1 - h) - \beta_h h \quad with \quad \alpha_h \frac{A(B - V)}{1 - e^{(V-B)/C}} \quad and \quad \beta_h \frac{A}{1 + e^{(B-V)/C}}$$

$$\frac{dn}{dt} = \alpha_n (1 - n) - \beta_n n \quad with \quad \alpha_n \frac{A(V - B)}{1 - e^{(B-V)/C}} \quad and \quad \beta_n \frac{A(B - V)}{1 - e^{(V-B)/C}}$$

A, B and C are constants and independently specified for $\propto$ and β of each channel. Ca^{2+} is treated differently, because Ca^{2+} pools are assumed to be inside the cell near the cell membrane and can activate Ca^{2+} gated K^+ channels to achieve hyperpolarization. Using q to represent Ca^{2+} activation, a relation similar to the Na^+ channel activation (m) holds:

$$\frac{dq}{dt} = \propto_q (1-q) - \beta_q q \quad with \quad \alpha_q \frac{A(V-B)}{1-e^{(B-V)/C}} \quad and \quad \beta_q \frac{A(B-V)}{1-e^{(V-B)/C}}$$

with the Ca^{2+} current into the cell being $I_{Ca} = \bar{g}_{Ca} q^5 (V - E_{Ca})$.

If we denote Ca^{2+} entering the cell as entering the Ca_{AP} pool, then the change in concentration of $[Ca_{AP}]$ is equivalent to the rate of ions entering the pool and less the ions leaving the pool: $\frac{d[Ca_{AP}]}{dt} = \varphi_{AP} q^5 (V - E_{Ca}) - \delta_{AP}[Ca_{AP}]$,

where φ_{AP} is the rate of Ca^{2+} influx and δ_{AP} is the rate of decay. The concentration $[Ca_{AP}]$ will activate Ca^{2+} gated K^+ channels inside the cell membrane with the following current:

$$I_{K_{Ca}} = \bar{g}_{K_{Ca}} (V - E_K)[Ca_{AP}]$$

After an increased neural firing rate, calcium buildup in the cell will cause hyperpolarization and a reduction in the firing rate.

Synaptic equations

For implementing the synaptic coupling, neurotransmitter gated ionotropic synapses were modeled, where the channels conduct ionic current produced by a voltage driving force and channel conductance. AMPA and $GABA_A$ currents are governed by:

$$I_{syn} = (E_{syn} - V) G_{syn} s \quad 0 \leq s \leq 1$$

Where s is the level of synaptic activation, with 1 being the most active. All synapses are consolidating and saturating as defined by Lytton [43] and depressing as defined by Varela et

al. [44]. Every synaptic spike results in neurotransmitter release for duration C_{dur} when it binds to receptors with binding rate $\propto$ and unbinding rate β. Saturation occurs because any spike following another spike by less than C_{dur} extends neurotransmitter release for another C_{dur} interval. W_{sum} is the sum of all synaptic weights currently active within C_{dur}. After each spike and during C_{dur}, W_{sum} is incremented by the synaptic weight W_{syn} and after C_{dur}, W_{sum} is decremented by W_{syn}. Consolidation occurs by summing across synaptic activations into state variables R_{on} and R_{off}, which have the following dynamics:

$$\frac{dR_{on}}{dt} = \frac{W_{sum}R_{inf} - R_{on}}{R_{tau}} \qquad \frac{dR_{off}}{dt} = -\beta R_{off} \qquad R_{inf} = \frac{\propto}{\propto + \beta}$$

The consolidated level of synaptic activation is represented by $s = R_{on} + R_{off}$. For synaptic depression, W_{syn} is decreased during C_{dur} according to recent short-term pre-synaptic activity with: $W_{syn} = W_{syn}d_{fast}d_{slow}$, where depression variable $d_i = d_i D_i$ after a spike occurs, which then decays to 1 with $d_i = 1 - (1 - d_i)e^{-t/\tau_i}$. NMDA synapses are similar to AMPA and $GABA_A$ but with additional dynamics for the Mg^{2+} block.

$$I_{NMDA} = (E_{NMDA} - V)G_{NMDA}ps \quad 0 \le s \le 1 \quad 0 \le p \le 1$$

Where p is the voltage gated variable for the Mg^{2+} block with the following dynamics:

$$\frac{dp}{dt} = \propto_p (1 - p) - \beta_p p \quad with \quad \alpha_p = A_{\propto} e^{\frac{V}{C}} \qquad \beta_p = Ae^{-V/C}$$

The constants A and C are independently specified for $\propto$ and β of channel p. Both the layer II/III pyramidal and basket cells receive noise input from an excitatory synapse driven by a 300 Hz Poisson process. The pyramidal cell has the noise synapse on the apical dendrite and the basket cell has it on the basal dendrite. The noise synapse is identical to the AMPA synapse but without synaptic depression, and a decay time constant of 10 ms. The synaptic

noise conductance G_{syn} is multiplied by a noise level coefficient u when increased in the presence of a tumor.

Table S1. Based on equations from Silverstein and Lansner [23], and described previously by Ekeberg et al. [42], and by Fransén and Lansner [45].

		**Na^+**	**Na^+**	**K^+**	**Ca^{2+}**	**NMDA**
		**M**	**H**	**N**	**q**	**p**
**α**	**A (mV^{-1} ms^{-1})**	0.58	0.232	0.058	0.232	2.03 ms^{-1}
	**B (mV)**	-50	-50	-50	10	-
	**C (mV)**	1	1	0.8	11	17
**β**	**A (mV^{-1} ms^{-1})**	0.174	1.16 (ms^{-1})	0.0145	0.0029	0.029 ms^{-1}
	**B (mV)**	-59	-46	-40	10	-
	**C (mV)**	20	2	0.4	0.5	17

Table S2. Neuron parameters

Parameter	Pyramidal	Basket	Unit
E_{leak}	-65	-65	mV
E_{Na}	50	50	mV
E_{Ca}	150	150	mV
E_K	-80	-80	mV
E_{CA}(NMDA)	20	20	mV
C_m	0.01	0.01	$\mu F/mm^2$
g_m	0.74	0.44	$\mu S/mm^2$
g_{ext}	0.082	0.15	$\mu S/mm^2$
g_{na} soma	$150 \pm 2\%$	150	$\mu S/mm^2$
g_k soma	$250 \pm 2\%$	1000	$\mu S/mm^2$
g_{na} initial segment	2500	2500	$\mu S/mm^2$
g_k initial segment	83	5010	$\mu S/mm^2$
Ca_v influx rate	1.00	1.00	$mV^{-1}ms^{-1}mm^{-2}$
Ca_{NMDA} influx rate	2.96	-	$s^{-1}mV^{-1}\mu S^{-1}$
Ca_{NMDA} decay rate	1	-	s^{-1}
Ca_v decay rate	6.3	-	s^{-1}
g_k (Ca_v)	29.4	0.368	nS
g_k (Ca_{NMDA})	40	-	nS
soma diameter $\pm$ stdev	21 ± 2.1	7 ± 0.7	μm
total compartments	4	3	
dendritic area (rel. soma)	4	4	
initial seg area (rel. soma)	0.1	0.1	

Table S3. Synaptic parameters

**Pre-Post**	**Type**	C_{dur} **sec**	τ_{raise} sec	τ_{decay} sec	E_{rev} mV	D_{fast}	D_{slow}	τ_{rec} ms	τ_{rec} ms	E_{slow} mV
Pyr-Pyr	Kainate/AMPA	0.0	0.0	0.006	0	0.78	0.97	634	9300	-
Pyr-Pyr	NMDA	0.02	0.005	0.150	0	0.78	0.97	634	9300	0.020
Pyr-Basket	Kainate/AMPA	0.0	0.0	0.006	0	0.78	0.97	634	9300	-
Basket-Pyr	$GABA_A$	0.0	0.0	0.006	-85	0.94	-	1900	-	-
Noise	Kainate/AMPA	0.0	0.0	0.01	0	-	-	-	-	-

Table S4. Synaptic conductances

Synapses	Type	Conductance
minicolumn (local) Pyr-Pyr	Kainate/AMPA	0.35 nS
minicolumn (local) Pyr-Pyr	NMDA	0.033 nS
hypercolumn Pyr-Basket	Kainate/AMPA	0.044 nS
hypercolumn Basket-Pyr	$GABA_A$	1.9 nS
patch (long-range) Pyr-Pyr	Kainate/AMPA	0.041 nS
patch (long-range) Pyr-Pyr	NMDA	0.0036 nS
projection (fiber) Pyr-Pyr	Kainate/AMPA	0.25 nS
Projection (fiber) Pyr-Pyr	NMDA	0.023 nS

REFERENCES

23) Silverstein, DN, Lansner A (2011) Is attentional blink a byproduct of neocortical attractors? Frontiers in Computational Neuroscience 5: 13.

41) Hodgkin AL, Huxley AF (1952) A quantitative description of membrane current and its application to conduction and excitation in nerve. J Physiol 117: 500-544.

42) Ekeberg Ö, Wallén P, Lansner A, Tråvén H, Brodin L, Grillner S (1991) A computer based model for realistic simulations of neural networks. Biol Cybern 65: 81-90.

43) Lytton W (1996) Optimizing synaptic conductance calculation for network simulations. Neural Comput 8: 501–509.

44) Varela J, Sen K, Gibson J, Fost J, Abbott L, Nelson S (1997) A qualitative description of short-term plasticity at excitatory synapses in layer 2/3 of rat primary visual cortex. J Neurosci 17: 7926–7940.

45) Fransén E, Lansner A (1998) A model of cortical associative memory based on a horizontal network of connected columns. Network 9: 235-264.
