# Supplementary figures and images for: Pathological Neural Attractor Dynamics in Slowly Growing Gliomas Supports an Optimal Time Frame for White Matter Plasticity
Source: PLoS One. 2013 Jul 26;8(7):e69798. doi: 10.1371/journal.pone.0069798 (PMC3724895; doi:10.1371/journal.pone.0069798)

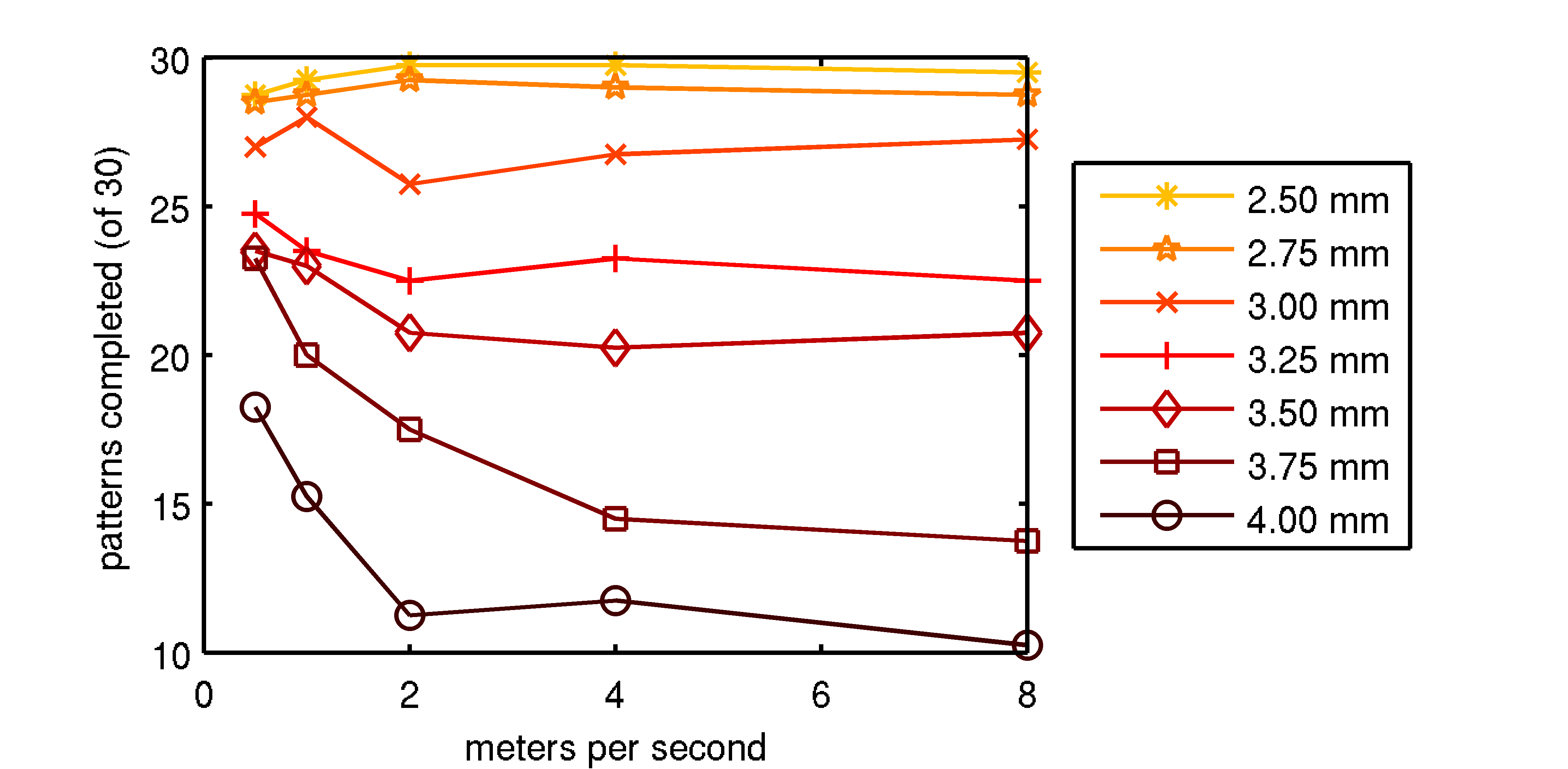

Supplement: Figure S1 — The effect of conduction velocity on network performance. Conduction velocity was varied between 0.5 m/s and 8 m/s and the network performance was quantified by the number of completed patterns. Seven different tumor radiuses were examined: 2.5 mm, 2.75 mm, 3 mm, 3.25 mm, 3.5 mm, 3.75 mm, 4.00 mm. With the larger tumor sizes, the network performance increased as the conduction velocity decreased. (TIF) [file pone.0069798.s002.tif]
